# Pathogenicity and virulence of Japanese encephalitis virus: Neuroinflammation and neuronal cell damage

**DOI:** 10.1080/21505594.2021.1899674

**Published:** 2021-03-16

**Authors:** Usama Ashraf, Zhen Ding, Shunzhou Deng, Jing Ye, Shengbo Cao, Zheng Chen

**Affiliations:** a State Key Laboratory of Agricultural Microbiology, Huazhong Agricultural University, Wuhan, Hubei, P. R. China; b The Cooperative Innovation Center for Sustainable Pig Production, Huazhong Agricultural University, Wuhan, Hubei, P. R. China; c Department of Preventive Veterinary Medicine, College of Animal Science and Technology, Jiangxi Agricultural University, Nanchang, Jiangxi, P. R. China; d Key Laboratory for Animal Health of Jiangxi Province, Nanchang, Jiangxi, P. R. China

**Keywords:** Japanese encephalitis, neuroinflammation, neuronal cell damage, glia, therapy

## Abstract

Thousands of human deaths occur annually due to Japanese encephalitis (JE), caused by Japanese encephalitis virus. During the virus infection of the central nervous system, reactive gliosis, uncontrolled inflammatory response, and neuronal cell death are considered as the characteristic features of JE. To date, no specific treatment has been approved to overcome JE, indicating a need for the development of novel therapies. In this article, we focused on basic biological mechanisms in glial (microglia and astrocytes) and neuronal cells that contribute to the onset of neuroinflammation and neuronal cell damage during Japanese encephalitis virus infection. We also provided comprehensive knowledge about anti-JE therapies tested in clinical or pre-clinical settings, and discussed recent therapeutic strategies that could be employed for JE treatment. The improved understanding of JE pathogenesis might lay a foundation for the development of novel therapies to halt JE.

**Abbreviations** AKT: a serine/threonine-specific protein kinase; AP1: activator protein 1; ASC: apoptosis-associated speck-like protein containing a CARD; ASK1: apoptosis signal-regulated kinase 1; ATF3/4/6: activating transcription factor 3/4/6; ATG5/7: autophagy-related 5/7; BBB: blood-brain barrier; Bcl-3/6: B-cell lymphoma 3/6 protein; CCL: C-C motif chemokine ligand; CCR2: C-C motif chemokine receptor 2; CHOP: C/EBP homologous protein; circRNA: circular RNA; CNS: central nervous system; CXCL: C-X-C motif chemokine ligand; dsRNA: double-stranded RNA; EDEM1: endoplasmic reticulum degradation enhancer mannosidase alpha-like 1; eIF2-ɑ: eukaryotic initiation factor 2 alpha; ER: endoplasmic reticulum; ERK: extracellular signal-regulated kinase; GRP78: 78-kDa glucose-regulated protein; ICAM: intercellular adhesion molecule; IFN: interferon; IL: interleukin; iNOS: inducible nitric oxide synthase; IRAK1/2: interleukin-1 receptor-associated kinase 1/2; IRE-1: inositol-requiring enzyme 1; IRF: interferon regulatory factor; ISG15: interferon-stimulated gene 15; JE: Japanese encephalitis; JEV: Japanese encephalitis virus; JNK: c-Jun N-terminal kinase; LAMP2: lysosome-associated membrane protein type 2; LC3-I/II: microtubule-associated protein 1 light chain 3-I/II; lncRNA: long non-coding RNA; MAPK: mitogen-activated protein kinase; miR/miRNA: microRNA; MK2: mitogen-activated protein kinase-activated protein kinase 2; MKK4: mitogen-activated protein kinase kinase 4; MLKL: mixed-linage kinase domain-like protein; MMP: matrix metalloproteinase; MyD88: myeloid differentiation factor 88; Nedd4: neural precursor cell-expressed developmentally downregulated 4; NF-κB: nuclear factor kappa B; NKRF: nuclear factor kappa B repressing factor; NLRP3: NLR family pyrin domain containing 3; NMDAR: N-methyl-D-aspartate receptor; NO: nitric oxide; NS2B/3/4: JEV non-structural protein 2B/3/4; P: phosphorylation. p38: mitogen-activated protein kinase p38; PKA: protein kinase A; PAK4: p21-activated kinase 4; PDFGR: platelet-derived growth factor receptor; PERK: protein kinase R-like endoplasmic reticulum kinase; PI3K: phosphoinositide 3-kinase; PTEN: phosphatase and tensin homolog; Rab7: Ras-related GTPase 7; Raf: proto-oncogene tyrosine-protein kinase Raf; Ras: a GTPase; RIDD: regulated IRE-1-dependent decay; RIG-I: retinoic acid-inducible gene I; RIPK1/3: receptor-interacting protein kinase 1/3; RNF11/125: RING finger protein 11/125; ROS: reactive oxygen species; SHIP1: SH2-containing inositol 5ʹ phosphatase 1; SOCS5: suppressor of cytokine signaling 5; Src: proto-oncogene tyrosine-protein kinase Src; ssRNA = single-stranded RNA; STAT: signal transducer and activator of transcription; TLR: toll-like receptor; TNFAIP3: tumor necrosis factor alpha-induced protein 3; TNFAR: tumor necrosis factor alpha receptor; TNF-α: tumor necrosis factor-alpha; TRAF6: tumor necrosis factor receptor-associated factor 6; TRIF: TIR-domain-containing adapter-inducing interferon-β; TRIM25: tripartite motif-containing 25; VCAM: vascular cell adhesion molecule; ZO-1: zonula occludens-1.

## Introduction

Japanese encephalitis (JE) is caused by mosquito-borne Japanese encephalitis virus (JEV), a positive-sense, single-stranded RNA virus, belonging to the genus *Flavivirus* of the family Flaviviridae [1]. JE is one of the most common forms of endemic encephalitis in humans and occasionally in animals around the globe, especially in the entire region of South, Southeast Asia, eastern regions of Russia, a few parts of Australia, and the Western Pacific islands (Saipan and Papua New Guinea) [2,3]. According to the World Health Organization report, the global estimate of JE incidence is ~68,000 cases per year, with roughly 13,600 to 20,400 deaths. Severe JE is characterized by high fever, headache, stiffness of neck muscles, disorientation, seizures, paralysis, coma, and eventually death. Albeit the majority of JE cases are asymptomatic or mild with fever and headache, a case-fatality rate of ~30% can be observed in those with encephalitis. Persistent neurological deficits, including paralysis, recurrent seizures, and speaking inability, can appear in 30% to 50% of patients presenting encephalitis. Young children display a high risk of getting severe JE, but individuals of any age can also be suffered [4]. Immunoprophylaxis is considered as the most effective method to prevent JE. Currently, four types of JEV vaccines have been approved for mass-scale immunization: live attenuated vaccine SA14-14-2, inactivated mouse brain-derived vaccine, inactivated Vero cell culture vaccine, and attenuated chimeric vaccine [5]. Despite the highly efficacious nature of these vaccines, the incidence of JE cases is still increasing [6], suggesting the need for the establishment of therapies to treat JEV infections.

The pathogenesis of JE, including neuroinvasion, neuroinflammation, and neuronal cell damage, is complicated; therefore, it has not yet been understood completely. The currently accepted narrative is that JEV gains access to the central nervous system (CNS) by breaching the blood-brain barrier (BBB), followed by stimulation of profound neuroinflammatory response in glial cells (microglia and astrocytes) and subsequent neuronal cell damage. In the past several years, efforts have been made in order to deeply understand the biology and pathogenesis of JE, and thus, highlighted several cellular and viral factors that facilitate the JEV replication and enhance the JEV-associated neuropathology. Herein, we reviewed the substantial research progress in understanding the basic mechanisms of JEV-induced neuroinflammation and neuronal cell damage, followed by a comprehensive overview of current therapeutic options to treat JE. Based on available knowledge, research gaps have also been highlighted that might be crucial to understand JE pathogenesis and to develop novel therapies for JE.

## Neuroinflammation in JE

### JEV replication in the periphery and neuroinvasion

**Figure 1.**
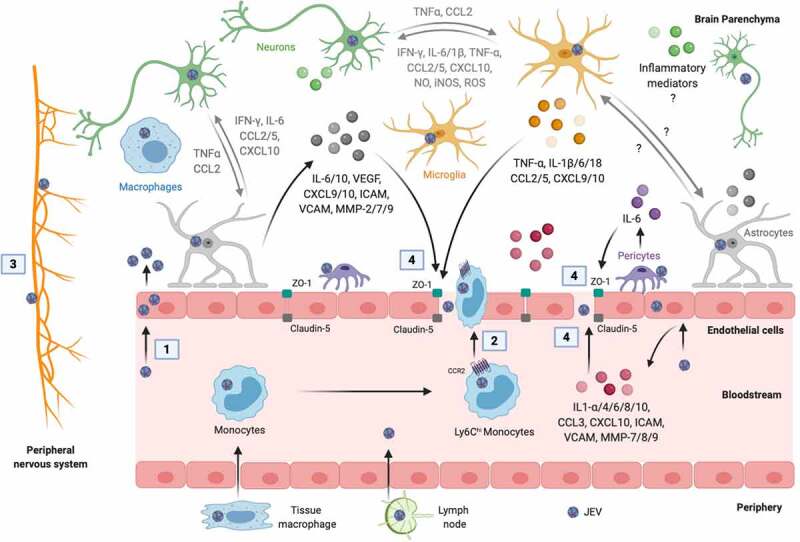
**Mechanisms of JEV neuroinvasion**. The numbers in squares indicate the mechanisms of virus entry into the brain: 1, passive transport of virus particles across the endothelial cells; 2, diapedesis of infected leukocytes; 3, virus transport via the peripheral nervous system; and 4, virus transport through the BBB disrupted by inflammatory mediators released from cells of blood and brain sides of the BBB. The inflammatory mediators written in gray mediate the crosstalk between microglia, astrocytes, and neurons that may contribute to the BBB damage. The symbol “?” denotes the missing information in the literature. Created with the web-based BioRender tool (BioRender.com)

An overview of JEV entry into the CNS is shown in **Figure 1**. Once an individual is bitten by a JEV-infected mosquito, the virus infects the resident dermal cells (dendritic cells, fibroblasts, endothelial cells, and pericytes) and local lymph nodes, followed by the onset of primary asymptomatic viremia [7]. Later, the virus disseminates via hematogenous route and efferent lymphatic system to multiple body organs (e.g., heart, liver, spleen, and muscle), causing secondary symptomatic viremia. In the periphery, JEV mainly replicates in the macrophage/dendritic cell precursor-derived Ly6C^hi^ monocytes that express abundant surface CCR2, and subsequently, can migrate from the periphery to the CNS and contribute to the inflammatory response [7,8].

The ingress of the virus in the CNS is indispensable for inducing neuroinflammation and neuronal cell damage. Several lines of evidence demonstrate that the virus reaches the CNS via crossing the BBB, which is comprised of brain microvasculature endothelial cells together with neurovasculature units (pericytes, astrocytes, microglia, neurons, and extracellular matrix), and maintains the CNS homeostasis by regulating the transport of immune cells and soluble molecules from blood to brain [9,10]. Endothelial cells in the brain microvasculature are considered as the key cells to provide structural and functional integrity to BBB. The interaction of neurovasculature cells with each other and their cooperation with endothelial cells is also vital for tight junctions and regulation of the BBB integrity [9,10]. The mechanisms by which JEV crosses the BBB to enter into the CNS are supported by four possible modes: (1) proliferation of virus within endothelial cells without affecting the cell viability, followed by passive transport of viral particles across the endothelial cells, (2) diapedesis of virus-infected leukocytes between endothelial cell junctions, (3) transport via the peripheral nervous system, and (4) disruption of the BBB through the release of virus-induced inflammatory mediators from the cells of apical (blood) and basolateral (brain) sides of the BBB, which is considered to be the most common mode [11,12].

Infection of endothelial cells by JEV caused no cytotoxicity through the deactivation of cellular pro-apoptotic proteins, but increased permeability of endothelial cells, excluding the apoptosis of endothelial cells as a cell process associated with the BBB disruption [13]. In an *in vitro* human BBB model, JEV has been shown to stimulate the release of inflammatory mediators (cytokines, chemokines, matrix metalloproteinases, and cellular adhesion molecules) from endothelial cells and astrocytes, which permit the virus entry into the brain by disrupting the BBB [14]. JEV-induced IL-6 production from cultured pericytes led to the activation of ubiquitin-proteasome and the degradation of ZO-1 in co-cultured endothelial cells, contributing to the BBB disruption [15]. Similarly, the treatment of cultured endothelial cells with IL-6, VEGF, MMP-2, and MMP-9, secreted from JEV-infected astrocytes, resulted in ubiquitin-proteasome activation, tight junction proteins (ZO-1 and claudin-5) proteasomal degradation, and subsequent endothelial cell barrier dysfunction via the stimulation of Janus kinase-2/STAT3 signaling and the induction of n-recognin-1 (ubiquitin-protein ligase E3 component) in endothelial cells [16]. In addition, activated microglia secrete inflammatory cytokines, which in turn promote the BBB leakage [17].

In contrast, JEV has also been observed to gain entry into the CNS prior to disrupting the BBB. Incubation of cultured monolayers of endothelial cells with brain extracts obtained from JEV-infected mice, but not with direct JEV particles, induced alterations in the permeability of the BBB through inflammatory mediators (CXCL10, CCL2/3/4, and IL-6) associated inhibition of tight junction proteins [18]. CXCL10 also affects the distribution of ZO-1 and claudin-5 in endothelial cells and subsequently promotes the BBB damage by augmenting the activity of TNF-α through the JNK signaling pathway in infected mice [19]. Thus, these data suggest that the infection of endothelial cells is not linked to barrier leakage. Further understanding of the mechanisms by which JEV breaches the BBB and the role of host immune response to mediate the BBB breakdown is required which may lead to the development of novel therapeutic approaches to prevent JE.

## Activation of glial and neuronal cells

**Figure 2.**
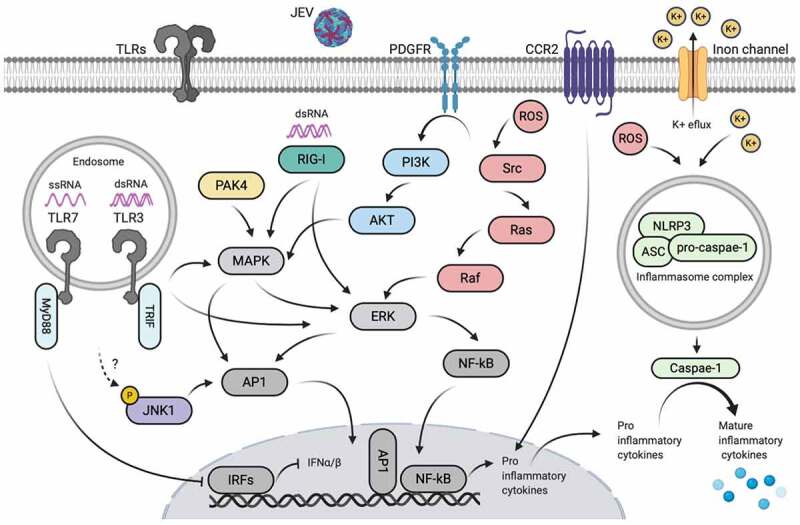
**Proteins-mediated active inflammatory responses during JEV infection**. The symbol “?” denotes the unidentified upstream regulator. Created with the web-based BioRender tool (BioRender.com)

**Figure 3.**
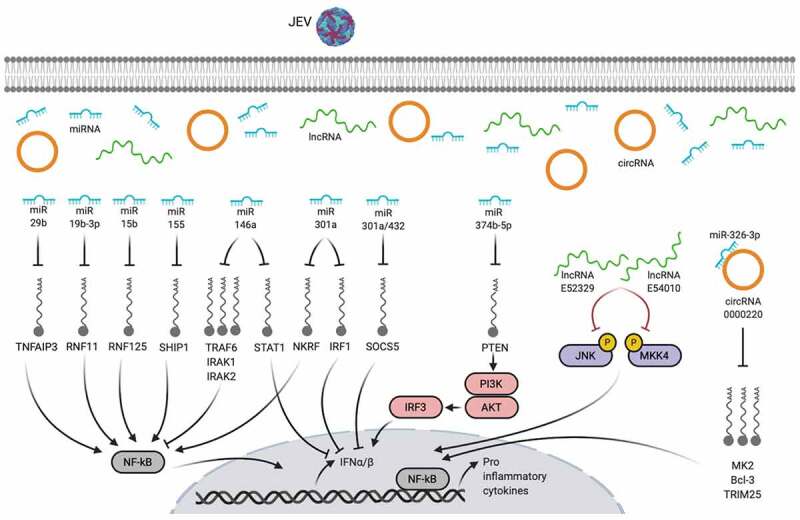
**Non-coding RNA-mediated active inflammatory responses during JEV infection**. Created with the web-based BioRender tool (BioRender.com)

Once the JEV entered the brain, the substantial activation of glial (microglia and astrocytes) and neuronal cells is a hallmark feature of JE. Microglia, astrocytes, and neurons have employed several mechanisms to trigger the neuroinflammatory response. It is well established that these cells can detect structurally conserved pathogen-associated molecular patterns by expressing a wide array to pattern recognition receptors, resulting in the stimulation of immune signaling cascades that lead to the production of inflammatory mediators. Several proteins (**Figure 2**) and non-coding RNA (**Figure 3**) entities have been shown to participate in the regulation of neuroinflammatory response in JE.

JEV infection of cultured mouse microglia resulted in the activation of TLR3- and RIG-I-mediated activation of ERK/MAPKp38/AP-1/NF-κB signaling cascades, leading to the production of inflammatory cytokines [20]. In the neuron/glial co-culture system, JEV induced a ROS-dependent activation of Src/Ras/Raf/ERK/NF-κB signaling axis [21]. The production of ROS and K^+^ efflux in JEV-infected cultured mouse microglia and mouse brain are observed to activate NLRP3 inflammasome signaling to form inflammasome complex, caspase-1 activation, and mature cytokines production [22]. The JEV-induced expression of CCR2 on the surface of microglia is positively correlated with the neurotoxic microglia activation phenotype and subsequent inflammatory response [23]. Regarding the astrocytic immune mechanisms, JEV infection of cultured human or mouse astrocytes is known to trigger PAK4/MAPK-NF-κB/AP-1 [24] and ROS/Src/PDGFR/PI3K/Akt/MAPK/AP-1 [25] signaling pathways, respectively, to enhance the production of inflammatory cytokines. The interaction between JEV subgenomic RNA (ssRNA) and TLR7, as assessed by co-immunoprecipitation, negatively regulated type-I IFN and inflammatory response in cultured neurons and mice brain tissues [26]. Moreover, the deficiency of TLR7 caused the stimulation of TLR8-mediated compensatory immune response in JEV-infected mice brain [27], suggesting that TLR7 and TLR8 are interlinked to mediate immune response during JEV infection. The role of TLRs, other than TLR3, TLR7, and TLR8, in regulating JEV-induced inflammatory responses in microglia, astrocytes, neurons, or brain, is currently unknown.

Global phosphoproteomics analysis of JEV-infected glial cells revealed alteration in the phosphorylation status of proteins mainly associated with cell division, signal transduction, transcription regulation, and cytoskeleton. Importantly, the JEV infection led to the overrepresentation of substrates of JNK1, together with increased activity of AKT1 and PKA signaling, followed by an elevated inflammatory response [28]. Furthermore, NOD1, belonging to the NOD-like receptors family, has been confirmed by our group to participate in orchestrating the inflammatory response during JEV infection (unpublished data), but the underlying mechanism is uncovered so far.

In addition to the direct modulation of immune response proteins in glia and neurons, the perturbation of the signatures of non-coding RNAs upon JEV infection in these cells has been associated with increased neuroinflammation. Of the non-coding RNAs, the functions of microRNAs (miRNAs) in regulating the neuroinflammatory response have been studied extensively in the last few years. Global RNA sequence profiling of JEV-infected mice brain tissues revealed differential expression of host-encoded miRNAs which were predicted to regulate antiviral immunity, apoptosis, neuronal differentiation, neurotrophin signaling, and transcription [29]. The increased expression of miR-29b, miR-19b-3p, miR-15b, and miR-301a in JEV-activated cultured microglia or astrocytes augmented the NF-κB activity via targeting the expression of TNFAIP3, RNF11, RNF125, and NKRF, respectively, leading to an increased inflammatory response [30–33]. The JEV-induced miR-155 positively regulated NF-κB activity by targeting SHIP1 in mouse microglial cells [34], whereas the same miRNA negatively regulated innate immune responses by attenuating IRF8 and NF-κB pathway mediators during JEV infection of human microglial cells [35], which indicates a species-specific mechanism. The inhibition of PTEN by miR-374b-5p modulated the PI3K/AKT/IRF3 pathway-dependent type-I IFN response in infected microglia [36]. The miR-146a, via targeting TRAF6, IRAK1, IRAK2, and STAT1, has been found to negatively regulate NF-κB and Janus kinase/STAT signaling in JEV JaOArS982 strain-infected human microglia, whereas, in the same cell model system, the JEV P20778 strain had opposite effects [37], highlighting a strain-dependent mechanism of neuropathogenesis. The ectopic expression of miR-432, known to be suppressed upon JEV infection, increased STAT1 phosphorylation through inhibiting SOCS5, which in turn, enhanced inflammatory responses and reduced virus replication in human microglial cells [38]. Moreover, the expression of some other brain-specific miRNAs (miR-21-5p, miR-342-3p, and miR-150-5p) during JEV infection has been predicted to control the transforming growth factor-β, MAPK, exon guidance, and nerve growth factor associated pathways [39]. Apart from glial cells, JEV infection is found to modulate the expression of miRNAs in neuronal cells. The miR-301a blocked the type-I IFN response and facilitated JE pathogenesis in mouse neuronal cells by suppressing the abundance of IRF1 and SOCS5 [40]. During the JEV infection of cultured neural stem/progenitor cells, several other miRNAs have been shown to differentially express, such as miR-9-5p (targets ETS1 and IL-6), miR-22-3p (targets MAX1, ESR1, NCOA1, and NR3C1), and miR-124-3p (targets CAV1, SDC4, IQGAP1, and PIK3CA) [41]. However, the effect of these miRNAs on predicted pathways remains void.

RNA sequence analyses of JEV-infected mice brain tissues have also unveiled widespread alterations in the expression of other non-coding RNAs that include long non-coding RNAs (lncRNAs) and circular RNAs (circRNAs). Functional enrichment analysis of differentially expressed lncRNAs and circRNAs exhibit a strong relationship with cellular processes related to innate immunity, inflammation, neurotransmission, and transcription dysregulation [42,43]. The siRNA-mediated silencing of lncRNAs E52329 and N54010 caused dephosphorylation of JNK and MKK4 proteins in cultured mouse microglia infected with JEV, resulting in the deactivation of JNK/MKK4 pathway and reduced inflammatory cytokines release [42]. Since circRNAs regulate mRNA expression via sponging miRNAs, the overexpression or knockdown of one of JEV-triggered circRNAs, circRNA-0000220, ameliorated inflammatory response in mouse microglial cells, which might have regulated through sponging the miR-326-3p and subsequently modulating the stability of miR-326-3p-target mRNAs, *viz*., MK2, Bcl-3, and TRIM25 [43]. Further studies providing deeper mechanistic insights into the functions of lncRNAs or circRNAs in mediating the neuroinflammatory response during JEV infection are required which may highlight potential therapeutic targets to treat JE.

Several kinds of inflammatory mediators are produced in response to the perturbation of the above-mentioned signaling proteins and non-coding RNA entities during JEV infection of microglia, astrocytes, and neurons, such as iNOS, TNF-α, IL-1β/6/12, CCL2/3/4/5, CXCL8/9/10/11, MMP-9, and IFN-α/β/γ. Once these mediators are released, they are subjected to initiate the secondary immune/inflammatory cascades, hence, exacerbating neuroinflammation and neuronal cell death [17]. The role of these inflammatory mediators in regulating the cross-talk between microglia, astrocytes, and neurons is sparsely known, and future studies in this research area may reveal novel mechanisms of neuroinflammation.

## Neuronal cell damage in JE

There have been several types of cell death modes described in the biomedical literature such as apoptosis, necroptosis, autophagy, pyroptosis, ferroptosis, phagocytosis, entosis, paraptosis, excitotoxicity, NETosis, and mitotic catastrophe [44,45]. Each of such modes is triggered and propagated by cellular mechanisms that show a considerable level of linkage with each other. Among them, only apoptosis, autophagy, necroptosis, and pyroptosis have been described with relevance to JE pathogenesis, as discussed below.

## Apoptosis

**Figure 4.**
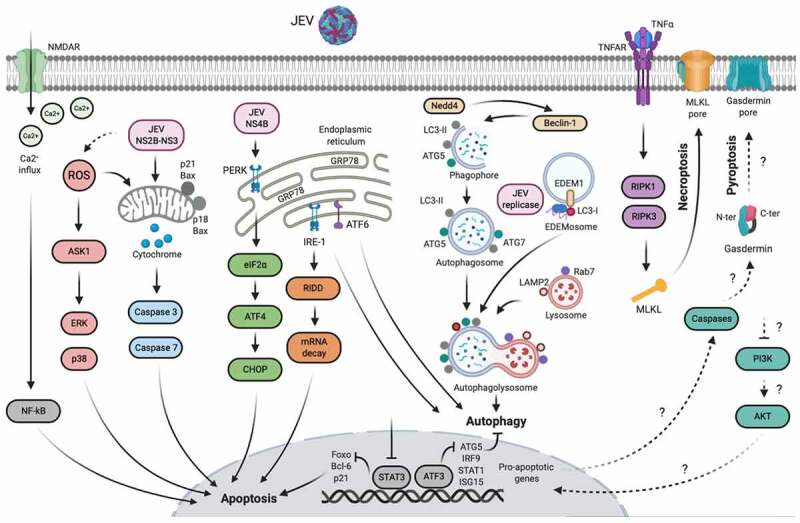
**Mechanisms of neuronal cell damage during JEV infection**. The symbol “?” denotes the cellular processes that have been confirmed in JEV-infected peritoneal macrophages, but not in neurons. Created with the web-based BioRender tool (BioRender.com)

The activation of glia or direct infection of neurons during JEV infection commences irreversible cellular responses, leading to neuronal apoptosis (**Figure 4**). Several lines of evidence have demonstrated the involvement of JEV-induced gliosis in establishing the inflammatory milieu that contributes to the onset of neuronal injury [46–48]. During the direct infection of neuronal cells, JEV can induce unfolded protein response by stressing the endoplasmic reticulum (ER) as assessed by the stimulation of CHOP, MAPK p38, and the IRE-1α-dependent decay pathway, which results in the apoptosis of neuronal cells [49,50]. A two-dimensional gel electrophoresis-based proteomic analysis of JEV-infected human neural stem/progenitor cells exhibits a dysregulated expression pattern of several ER stress response-related proteins, including GRP78, indicating sustained ER stress to induce apoptosis [51]. Another recent study demonstrated that JEV, through the interaction of its NS4B protein with an ER-resident stress sensor protein PERK, activates neuronal apoptosis both *in vitro* and *in vivo* by triggering the PERK/eIF2-α/ATF4/CHOP apoptosis pathway. Precisely, both the LIG-FHA and LIG-WD40 domains of NS4B protein were observed to induce the PERK dimerization, indicating that JEV NS4B protein pulls two PERK molecules together by interacting with them through different motifs. Furthermore, the expression of NS4B alone was sufficient to induce encephalitis in mice via PERK [52]. An integrated transcriptomic analysis of infected cultured neurons and mice brain tissues revealed the induction of neuronal apoptosis via impeding the STAT3/Foxo/Bcl-6/p21 pathway [53]. Furthermore, the involvement of mitochondrion-dependent pathways in regulating JEV-induced neuronal apoptosis has also been elucidated. Infection of cultured neuronal cells caused the JEV NS2B-NS3 protease-induced or p21-Bax/p18-Bax-mediated release of mitochondrial cytochrome C in the cytoplasm, indispensable for the activation of caspase-3/7-mediated apoptosis pathways in these cells [54,55]. Alternatively, the production of ROS and the stimulation of ASK1/ERK1/p38-MAPK pathway in infected cells is also linked to the NS4B-NS3-induced mitochondrion-dependent apoptosis [54,56]. The activation/phosphorylation of NMDAR is also observed as a pathogenic mechanism during JEV of cultured neurons and mice brain tissues, resulting in increased calcium ion influx in infected neurons and enhanced neuronal toxicity [46].

The apoptosis of JEV-infected cells represses virus replication and releases damage-associated molecular patterns which subsequently stimulate the innate and adaptive immune responses to trigger virus restriction and cellular debris clearance. How to augment virus restriction and cellular debris clearance is a key problem that needs to be understood.

## Autophagy

Apart from maintaining cellular homeostasis, autophagy is known to play a pivotal role during the replication of several viruses, including JEV (**Figure 4**). The propagation of some viruses is suppressed by autophagy, whereas other viruses can harness autophagy pathways to aid their replication [57]. Autophagy positively regulates JEV replication by regulating the early step of virus replication, i.e., entry and virus-uncoating [58]. JEV infection induced the accumulation of autophagy marker protein LC3-II in mouse neuronal cells and enhanced autophagosomes/autolysosomes in mice brains [59]. The depletion of autophagy-related genes (Rab7, LAMP2, ATG5, and Beclin-1) in JEV-infected mouse or human neuronal cells displayed cellular apoptosis and suppressed virus replication, suggesting that JEV employes autophagy as an immune evasion mechanism [58,59]. In addition, a recent study demonstrated a decisive association between autophagy and ER stress response pathways during JEV infection of neurons. The siRNA-mediated inhibition of IRE1- and ATF6-mediated ER stress pathways, but not PERK/eIF2-α-mediated pathway, curbed autophagy and escalated cellular apoptosis during JEV infection in mouse neuronal cells [60].

In contrast to the above findings, a few reports suggested a negative correlation between autophagy and JEV replication, indicating autophagy as a host-defensive mechanism against JEV. The production of JEV infectious particles was observed to be significantly increased in mouse neuronal cell deficient in ATG7 protein, and these cells were highly susceptible to virus-induced cellular apoptosis. Precisely, it was shown that JEV replication complexes (NS1 protein and replication intermediate dsRNA) colocalize with LC3-I and a marker of ER-associated degradation pathway, EDEM1, which suggests that JEV replication seems to take place on EDEMosomes comprised of LC3-I-positive and ERAD-originated EDEM1 membranous structures [61]. ATF3, induced following JEV infection, binds to the promoter sequence of ATG5, IRF9, STAT1, and ISG15 genes, thereby inhibiting autophagy and antiviral response to favor virus replication [62]. Furthermore, the JEV-induced expression of Nedd4 (E3 ubiquitin ligase) in human neuronal cells, but not in non-neuronal cells, promoted JEV replication by suppressing the autophagy as assessed by increased autophagosome accumulation in Nedd4-silenced cells, thus, implying it as a neuron-specific cellular mechanism [63].

Since the interplay between JEV and autophagy is currently known only in neuronal cells, investigating similar mechanisms in glial cells would be intriguing and may provide novel insights into the JEV-induced neuronal cell damages.

## Necroptosis and pyroptosis

Necroptosis and pyroptosis are relatively new forms of inflammatory programmed cell death. Necroptosis is triggered by death receptors, extracellular and intracellular DNA or RNA detecting sensors, IFNs, or some other stimuli through necrosomes incorporating RIPK1, RIPK3, and the substrate MLKL. Activation/phosphorylation of MLKL by RIPK3 caused aberrant flux of sodium and calcium ions and subsequent pore formation at the plasma membrane, thus, implying MLKL as an executor of necroptosis [64,65]. Pyroptosis is induced by inflammatory caspases, viz., mouse caspase 1/11 and human caspase 4/5, which initiate pyroptosis by proteolytically processing the gasdermin D protein, generating an N-terminal fragment that perforates the plasma membrane, leading to cell rupture [66]. The physical rupture of cells leads to the release of inflammatory cytokines and damage-associated molecular patterns, intensifying the inflammatory potential of necroptosis and pyroptosis. Both these processes, as host-defensive mechanisms, are employed by the host cell to control pathogenic infections, including those caused by viruses [66–68].

An overview of necroptosis and pyroptosis triggered during JE is shown in **Figure 4**. During JE, the MLKL-mediated necroptosis has been found in cultured neuronal cells and mice brain tissues as observed by immune-electron microscopy and immunochemistry. Inhibition of MLKL attenuated the production of inflammatory cytokines, reduced the onset of JE progression, and improved lethality in infected mice. The precise mechanism underlying the MLKL-induced necroptosis during JE is currently unexplored [69]. TNF-α is a crucial stimulator of necroptosis via TNF-α/TNFR1/RIPK1/RIPK3/MLKL pathway in several brain diseases [70,71]. Since TNF-α is also one of the main mediators of JE, it could be speculated that MLKL-induced necroptosis during JE may have occurred through the TNF-α-mediated mechanism; however, it needs to be confirmed by experimental approaches. In a mice model of JEV infection, the deficiency of Axl, a receptor tyrosine kinase, enhanced pyroptosis in 80% JEV-infected peritoneal macrophages by dampening the PI3K/Akt signaling pathway. The pyroptotic macrophages released a significant amount of IL-1α in the serum, which in turn, mediated JEV neuroinvasion by breaching the BBB and induced JE in mice [72]. Furthermore, using an in situ JEV-infection model system, the transcriptomic profiling of peritoneal macrophages revealed significant enrichment of programmed cell death pathways, including necroptosis and pyroptosis verified by immunostaining of specific markers, i.e., RIPK1 and gasdermin D [73].

In general, little is known regarding the role of necroptosis and pyroptosis during JE; therefore, studying these cellular processes extensively in neurons and glial cells may contribute significantly to the respective research field. Moreover, understanding the cross-talk between different pathways of programmed cells death could provide deep insights into the pathogenesis of JE.

## Therapeutic interventions to treat JE

Given the potential severity of JE, there is no specific treatment that can be prescribed to treat this disease. Currently, the only option to manage JE is the use of supportive treatment that can ameliorate clinical signs and support patients to overcome the infection. To date, despite productive research efforts, only five therapies have been entered into randomized clinical trials: dexamethasone (anti-inflammatory), IFNα2a (anti-viral), ribavirin (anti-viral), intravenous immunoglobulins (virus-neutralizing and anti-inflammatory), and minocycline (anti-inflammatory) [74–78]. None of the tested therapies affect the outcome of JE in patients and exhibited a non-significant difference when compared with the placebo control group. The therapeutic options that have shown potential efficacy against JEV during the pre-clinical studies in mice model of JEV infection include monoclonal antibodies 503 (JEV E protein-specific) and 2B8 (JEV NS1 protein-specific), pentoxifylline (TNF-α inhibitor), fenofibrate (NF-κB inhibitor), etanercept (TNF-α inhibitor), PGL5001 (JNK inhibitor), MK-801 (NMDAR inhibitor), and artemisinin (type-I IFN agonist), and some nonspecific anti-inflammatory and/or antiviral drugs (rosmarinic acid, arctigenin, and nitazoxanide) [28,46,79–87]. Of these, the only arctigenin displayed 100% efficacy with no mortality in JEV challenged mice, whereas all other therapies showed variable mortality with a survival rate ranging from 40% to 80%. In addition, ivermectin (JEV NS3 inhibitor), decanoyl-Arg-Val-Lys-Arg-chloromethylketone (furin-mediated JEV prM-to-M cleavage inhibitor), FGIN-1-27, niclosamide, and cilnidipine have demonstrated anti-JEV activity *in vitro* [88–90].

RNA-based therapeutics have also shown promising effects against JE. Treatment of JEV-infected mice with antagomirs specific for miR-19b-3p, miR-15b, miR-301a, and miR-155 ameliorated features of acute JE and improved behavior and survival rate of mice [31–34,40]. Alternatively, the infection of mice with an infectious recombinant JEV, incorporating two copies of the neuron-specific miR-124-recognition sequence into the 3ʹ untranslated region of the genome, elicited protective immunity against subsequent JEV challenge [91]. The use of single miRNA-like polycistrons, bearing a single RNA polymerase II promoter and simultaneously expressing siRNAs targeting conserved regions of JEV strains of four genotypes, showed highly effective and broad-spectrum anti-JEV activity *in vitro* [92]. Such types of therapeutic approaches could be applied for the establishment of effectual anti-JE or anti-JEV gene therapies in clinical settings.

Recently, a variety of nanostructures, including metallic or silica nanoparticles, graphene oxide, nanostructured glycans, and carbon dots, are found as novel and highly effective antiviral agents against several viruses. The multivalent structural features of nanoparticles allow them to interfere with different stages of virus life cycles: virus attachment to the host cells, viral RNA synthesis, and budding of newly-formed viral particles [93]. The non-cytotoxic benzoxazine monomer-derived carbon dots have demonstrated JEV infection-blocking activity *in vitro* through interacting with the surface of JEV particles, thereby obstructing the virus entry into the host cell [94]. Detailed experiments are required to examine whether the prophylactic or therapeutic implication of the nanoparticles against JEV will be suitable *in vivo*.

Considering the high rate of attrition and slow process of novel drug discovery, the drug repurposing is an efficient, low-budget, riskless, and time-saving strategy alternative to the traditional drug discovery process where existing drugs, having already being tested safe in humans, are redirected based on a valid target molecule to treat diseases. Hence, the therapeutic value of a drug is maximized and the success rate is increased consequently [95]. This approach, accompanied by modern methods of drug discovery, has been successfully employed against several pathologies, including viral infections [96,97]. Therefore, identifying the potential repurposed drugs may lead to the establishment of specific therapies to treat JE.

## Conclusions

JE results in high morbidity and mortality in humans with no specific treatment. The unavailability of specific treatment for JE is partly due to an incomplete understanding of the disease pathology. Research progress made in recent years has advanced our understanding of the basic mechanisms of JEV-induced neuroinflammation and neuronal cell damage, and has highlighted new therapeutic targets to treat JE. Several therapeutic options that have shown promising results in pre-clinical mice studies could be employed for clinical trials immediately. Given the extensive role of the innate immune system in the progression of JE, a combination treatment approach comprising both anti-inflammatory and antiviral drugs may provide a synergistic effect to treat clinical cases of JE in the future.

## Data Availability

Not applicable.

## Declarations

**Ethics approval and consent to participate**

Not applicable.
